# Supplementary material for: Loss of cultural song diversity and the convergence of songs in a declining Hawaiian forest bird community
Source: R Soc Open Sci. 2019 Aug 14;6(8):190719. doi: 10.1098/rsos.190719 (PMC6731710; doi:10.1098/rsos.190719)

Supplemental Figure S1

Figure S1. Representative ‘akeke‘e song spectrogram displaying the 11 measured acoustic variables: 1) song length (seconds), 2) total number of syllables (n=11), 3) number of unique syllable types (n=2), 4) trill rate (total syllables/song length), 5) average number of notes per syllable (n=1.5), 6) average number of frequency changes within a syllable (n=1.6), 7) number of frequency changes between syllables within the song (n=2), 8) peak frequency (kHz), 9) low (kHz) and 10) high (kHz) frequencies at which the amplitude exceeded -24dB relative to the peak, and 11) frequency bandwidth (kHz).

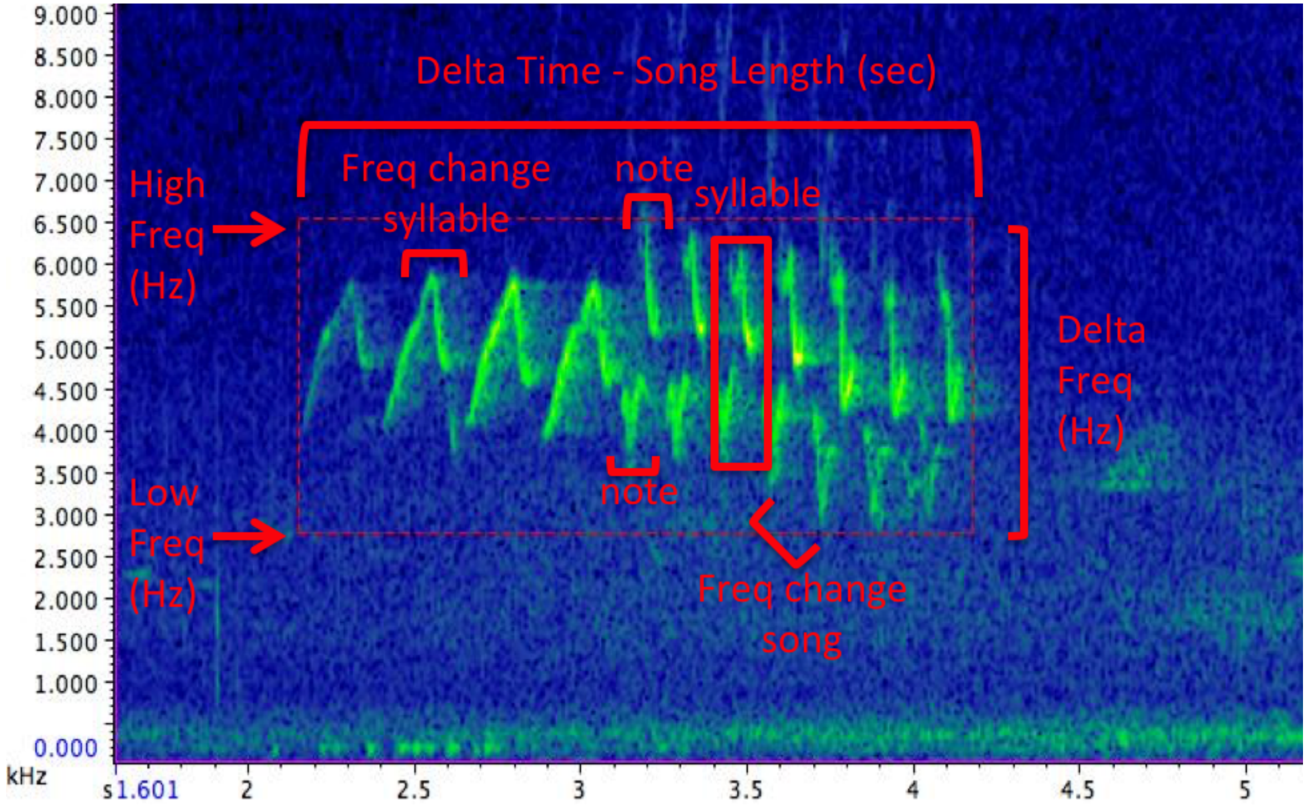

Supplement: Figure S1 [file rsos190719supp1.pdf]
