# Supplementary material for: Loss of cultural song diversity and the convergence of songs in a declining Hawaiian forest bird community
Source: R Soc Open Sci. 2019 Aug 14;6(8):190719. doi: 10.1098/rsos.190719 (PMC6731710; doi:10.1098/rsos.190719)
Supplement: Table S1 [file rsos190719supp2.pdf]

Supplemental Table S1

Table S1. Detailed information about each Kaua‘i ‘amakihi, ‘anianiau, and ‘akeke‘e recording including: unique identifier, date, location, and recordist, and type of recording equipment used to record each vocalization (recorder, microphone, and accessories). The unique identifier (Record ID) is the catalogue number given to each recording archived at The Macaulay Library at the Cornell Lab of Ornithology.

Record ID	Species	Time Period	Recordist	Date	Location	Recorder	Microphone	Accessory
ML5814	Kaua‘i ‘amakihi	1970s	Pratt, H. Douglas	1\30\1978	Koke‘e State Park	UHER 4200	Dan Gibson unspecified model	Parabola 18 in
ML5821	Kaua‘i ‘amakihi	1970s	Pratt, H. Douglas	1\30\1978	Honopu Trail in Koke‘e State Park	UHER 4200	Dan Gibson unspecified model	Parabola 18 in
ML5832	Kaua‘i ‘amakihi	1970s	Pratt, H. Douglas	1\31\1978	3.0 km E. Of Koke‘e State Park	UHER 4200	Dan Gibson unspecified model	Parabola 18 in
ML5022	Kaua‘i ‘amakihi	1970s	Pratt, H. Douglas	5\12\1976	Pu‘u Kila Lookout in Koke‘e State Park	SONY TC-45	Sony F16	Sony Parabola
ML5026	Kaua‘i ‘amakihi	1970s	Pratt, H. Douglas	5\12\1976	Pu‘u Kila Lookout in Koke‘e State Park	SONY TC-45	Sony F16	Sony Parabola
ML5028	Kaua‘i ‘amakihi	1970s	Pratt, H. Douglas	5\12\1976	Pu‘u Kila Lookout in Koke‘e State Park	SONY TC-45	Sony F16	Sony Parabola
ML5032	Kaua‘i ‘amakihi	1970s	Pratt, H. Douglas	5\12\1976	Pu‘u Kila Lookout in Koke‘e State Park	SONY TC-45	Sony F16	Sony Parabola
ML5035	Kaua‘i ‘amakihi	1970s	Pratt, H. Douglas	5\12\1976	Pu‘u Kila Lookout in Koke‘e State Park	SONY TC-45	Sony F16	Sony Parabola
ML5056	Kaua‘i ‘amakihi	1970s	Pratt, H. Douglas	5\14\1976	Road to Mōhihi in Koke‘e State Park	SONY TC-45	Sony F16	Sony Parabola

ML5109	Kaua‘i ‘amakihi	1970s	Pratt, H. Douglas	5\21\1977	Koke‘e State Park	SONY TC-45	Dan Gibson unspecified model	Parabola 18 in
ML5112	Kaua‘i ‘amakihi	1970s	Pratt, H. Douglas	5\21\1977	Koke‘e State Park	SONY TC-45	Dan Gibson unspecified model	Parabola 18 in
ML5102	Kaua‘i ‘amakihi	1970s	Pratt, H. Douglas	6\2\1977	Koke‘e State Park	SONY TC-45	Dan Gibson unspecified model	Parabola 18 in
ML5113	Kaua‘i ‘amakihi	1970s	Pratt, H. Douglas	6\2\1977	Koke‘e State Park	SONY TC-45	Dan Gibson unspecified model	Parabola 18 in
ML5115	Kaua‘i ‘amakihi	1970s	Pratt, H. Douglas	6\2\1977	Koke‘e State Park	SONY TC-45	Dan Gibson unspecified model	Parabola 18 in
ML5116	Kaua‘i ‘amakihi	1970s	Pratt, H. Douglas	6\2\1977	Koke‘e State Park	SONY TC-45	Dan Gibson unspecified model	Parabola 18 in
ML5118	Kaua‘i ‘amakihi	1970s	Pratt, H. Douglas	6\2\1977	Koke‘e State Park	SONY TC-45	Dan Gibson unspecified model	Parabola 18 in
ML5024	‘anianiau	1970s	Pratt, H. Douglas	5\12\1976	Pu‘u Kila Lookout in Koke‘e State Park	SONY TC-45	Sony F16	Sony Parabola
ML5025	‘anianiau	1970s	Pratt, H. Douglas	5\12\1976	Pu‘u Kila Lookout in Koke‘e State Park	SONY TC-45	Sony F16	Sony Parabola
ML5039	‘anianiau	1970s	Pratt, H. Douglas	5\13\1976	Pu‘u Kila Lookout in Koke‘e State Park	SONY TC-45	Sony F16	Sony Parabola
ML5047	‘anianiau	1970s	Pratt, H. Douglas	5\13\1976	Koke‘e State Park	SONY TC-45	Sony F16	Sony Parabola
ML5051	‘anianiau	1970s	Pratt, H. Douglas	5\13\1976	Koke‘e State Park	SONY TC-45	Sony F16	Sony Parabola
ML5052	‘anianiau	1970s	Pratt, H. Douglas	5\13\1976	Koke‘e State Park	SONY TC-45	Sony F16	Sony Parabola
ML5057	‘anianiau	1970s	Pratt, H. Douglas	5\14\1976	Road to Kumuwela in Koke‘e State Park	SONY TC-45	Sony F16	Sony Parabola

ML5120	‘anianiau	1970s	Pratt, H. Douglas	6\2\1977	Koke‘e State Park	SONY TC-45	Dan Gibson unspecified model	Parabola 18 in
ML5038	‘akeke‘e	1970s	Pratt, H. Douglas	5/13/1976	Pu‘u Kila Lookout in Koke‘e State Park	SONY TC-45	Sony F16	Sony Parabola
ML156095061	Kaua‘i ‘amakihi	Early 2000s	Kuhn, David	7/11/2001	Pihea Trail in Koke‘e State Park	Sony Minidisc	Telinga PRO-8 MK2	Telinga Parabola 22 in
ML156094711	Kaua‘i ‘amakihi	Early 2000s	Kuhn, David	7/15/2001	Pihea Trail in Koke‘e State Park	Sony Minidisc	Telinga PRO-8 MK2	Telinga Parabola 22 in
ML156094521	Kaua‘i ‘amakihi	Early 2000s	Kuhn, David	7/15/2001	Pihea Trail in Koke‘e State Park	Sony Minidisc	Telinga PRO-8 MK2	Telinga Parabola 22 in
ML156094151	Kaua‘i ‘amakihi	Early 2000s	Kuhn, David	2/13/2002	Pihea Trail in Koke‘e State Park	Sony Minidisc	Telinga PRO-8 MK2	Telinga Parabola 22 in
ML156092401	Kaua‘i ‘amakihi	Early 2000s	Kuhn, David	4/11/2002	Pihea Trail in Koke‘e State Park	Sony Minidisc	Telinga PRO-8 MK2	Telinga Parabola 22 in
ML156091191	Kaua‘i ‘amakihi	Early 2000s	Kuhn, David	4/8/2003	Pihea Trail in Koke‘e State Park	Sony Minidisc	Telinga PRO-8 MK2	Telinga Parabola 22 in
ML156096611	‘anianiau	Early 2000s	Kuhn, David	2004	Pihea Trail in Koke‘e State Park	Sony Minidisc	Telinga PRO-8 MK2	Telinga Parabola 22 in
ML156095391	‘anianiau	Early 2000s	Kuhn, David	4/15/2001	Pihea Trail in Koke‘e State Park	Sony Minidisc	Telinga PRO-8 MK2	Telinga Parabola 22 in
ML156095241	‘anianiau	Early 2000s	Kuhn, David	4/15/2001	Pihea Trail in Koke‘e State Park	Sony Minidisc	Telinga PRO-8 MK2	Telinga Parabola 22 in
ML156091971	‘anianiau	Early 2000s	Kuhn, David	4/15/2002	Pihea Trail in Koke‘e State Park	Sony Minidisc	Telinga PRO-8 MK2	Telinga Parabola 22 in
ML156091641	‘anianiau	Early 2000s	Kuhn, David	4/18/2002	Pihea Trail in Koke‘e State Park	Sony Minidisc	Telinga PRO-8 MK2	Telinga Parabola 22 in
ML156091651	‘anianiau	Early 2000s	Kuhn, David	4/18/2002	Pihea Trail in Koke‘e State Park	Sony Minidisc	Telinga PRO-8 MK2	Telinga Parabola 22 in
ML156091481	‘anianiau	Early 2000s	Kuhn, David	5/15/2002	Pihea Trail in Koke‘e State Park	Sony Minidisc	Telinga PRO-8 MK2	Telinga Parabola 22 in

ML156091201	‘anianiau	Early 2000s	Kuhn, David	4/8/2003	Pihea Trail in Koke‘e State Park	Sony Minidisc	Telinga PRO-8 MK2	Telinga Parabola 22 in
ML156091021	‘anianiau	Early 2000s	Kuhn, David	4/29/2003	Pihea Trail in Koke‘e State Park	Sony Minidisc	Telinga PRO-8 MK2	Telinga Parabola 22 in
ML156096151	‘akeke‘e	Early 2000s	Kuhn, David	4/2/2001	Pihea Trail in Koke‘e State Park	Sony Minidisc	Telinga PRO-8 MK2	Telinga Parabola 22 in
ML156095811	‘akeke‘e	Early 2000s	Kuhn, David	4/3/2001	Pihea Trail in Koke‘e State Park	Sony Minidisc	Telinga PRO-8 MK2	Telinga Parabola 22 in
ML156093291	‘akeke‘e	Early 2000s	Kuhn, David	2/28/2002	Pihea Trail in Koke‘e State Park	Sony Minidisc	Telinga PRO-8 MK2	Telinga Parabola 22 in
ML156093531	‘akeke‘e	Early 2000s	Kuhn, David	2/28/2002	Pihea Trail in Koke‘e State Park	Sony Minidisc	Telinga PRO-8 MK2	Telinga Parabola 22 in
ML156092881	‘akeke‘e	Early 2000s	Kuhn, David	4/3/2002	Pihea Trail in Koke‘e State Park	Sony Minidisc	Telinga PRO-8 MK2	Telinga Parabola 22 in
ML156092881	‘akeke‘e	Early 2000s	Kuhn, David	4/3/2002	Pihea Trail in Koke‘e State Park	Sony Minidisc	Telinga PRO-8 MK2	Telinga Parabola 22 in
ML156093021	‘akeke‘e	Early 2000s	Kuhn, David	4/3/2002	Pihea Trail in Koke‘e State Park	Sony Minidisc	Telinga PRO-8 MK2	Telinga Parabola 22 in
ML15609277	‘akeke‘e	Early 2000s	Kuhn, David	4/3/2002	Pihea Trail in Koke‘e State Park	Sony Minidisc	Telinga PRO-8 MK2	Telinga Parabola 22 in
ML156093011	‘akeke‘e	Early 2000s	Kuhn, David	4/3/2002	Pihea Trail in Koke‘e State Park	Sony Minidisc	Telinga PRO-8 MK2	Telinga Parabola 22 in
ML156092751	‘akeke‘e	Early 2000s	Kuhn, David	4/3/2002	Pihea Trail in Koke‘e State Park	Sony Minidisc	Telinga PRO-8 MK2	Telinga Parabola 22 in
ML156091921	‘akeke‘e	Early 2000s	Kuhn, David	4/15/2002	Pihea Trail in Koke‘e State Park	Sony Minidisc	Telinga PRO-8 MK2	Telinga Parabola 22 in
ML156090831	‘akeke‘e	Early 2000s	Kuhn, David	2/19/2004	Pihea Trail in Koke‘e State Park	Sony Minidisc	Telinga PRO-8 MK2	Telinga Parabola 22 in
ML156004801	Kaua‘i ‘amakihi	Present day	Kuhn, David	6/9/2010	Halepa'akai Stream	Sony PCM M10	Telinga PRO-8 MK2	Telinga Parabola 22 in

ML156002671	Kaua‘i ‘amakihi	Present day	Kuhn, David	6/10/2010	Halepa'akai Stream	Sony PCM M10	Telinga PRO-8 MK2	Telinga Parabola 22 in
ML155997081	Kaua‘i ‘amakihi	Present day	Kuhn, David	2/9/2014	Upper Kawaikōī Stream	Sony PCM M10	Telinga PRO-8 MK2	Telinga Parabola 22 in
ML155988551	Kaua‘i ‘amakihi	Present day	Kuhn, David	5/21/2014	Pihea Trail in Koke‘e State Park	Sony PCM M10	Telinga PRO-8 MK2	Telinga Parabola 22 in
ML155986081	Kaua‘i ‘amakihi	Present day	Kuhn, David	3/10/2015	Pihea Trail in Koke‘e State Park	Sony PCM M10	Telinga PRO-8 MK2	Telinga Parabola 22 in
ML155983261	Kaua‘i ‘amakihi	Present day	Kuhn, David	3/11/2015	Pihea Trail in Koke‘e State Park	Sony PCM M10	Telinga PRO-8 MK2	Telinga Parabola 22 in
ML155971191	Kaua‘i ‘amakihi	Present day	Hite, Justin	5/11/2015	Halepa'akai Stream	Marantz PMD 661	Sennheiser MKH 20	Telinga Parabola 15.5 in
ML155551431	Kaua‘i ‘amakihi	Present day	Kuhn, David	2/27/2017	Halepa'akai Stream	Sony PCM M10	Telinga PRO-8 MK2	Telinga Parabola 22 in
ML155551481	Kaua‘i ‘amakihi	Present day	Kuhn, David	2/27/2017	Halepa'akai Stream	Sony PCM M10	Telinga PRO-8 MK2	Telinga Parabola 22 in
ML155551441	Kaua‘i ‘amakihi	Present day	Paxton, Kristina	2/27/2017	Halepa'akai Stream	Marantz PMD 661	Sennheiser MKH 20	
ML155551411	Kaua‘i ‘amakihi	Present day	Paxton, Kristina	2/27/2017	Halepa'akai Stream	Marantz PMD 661	Sennheiser MKH 20	
ML155551451	Kaua‘i ‘amakihi	Present day	Paxton, Kristina	2/27/2017	Halepa'akai Stream	Marantz PMD 661	Sennheiser MKH 20	
ML155993031	‘anianiau	Present day	Kuhn, David	2/13/2014	Halepa'akai Stream	Sony PCM M10	Telinga PRO-8 MK2	Telinga Parabola 22 in
ML155979511	‘anianiau	Present day	Hite, Justin	3/28/2015	Halepa'akai Stream	Marantz PMD 661	Sennheiser MKH 20	Telinga Parabola 15.5 in
ML155981941	‘anianiau	Present day	Hite, Justin	3/28/2015	Halepa'akai Stream	Marantz PMD 661	Sennheiser MKH 20	Telinga Parabola 15.5 in
ML155977701	‘anianiau	Present day	Hite, Justin	3/29/2015	Halepa'akai Stream	Marantz PMD 661	Sennheiser MKH 20	Telinga Parabola 15.5 in

ML155551621	‘anianiau	Present day	Kuhn, David	2/26/2017	Halepa'akai Stream	Sony PCM M10	Telinga PRO-8 MK2	Telinga Parabola 22 in
ML15555157	‘anianiau	Present day	Kuhn, David	2/26/2017	Halepa'akai Stream	Sony PCM M10	Telinga PRO-8 MK2	Telinga Parabola 22 in
ML155551331	‘anianiau	Present day	Kuhn, David	2/27/2017	Halepa'akai Stream	Sony PCM M10	Telinga PRO-8 MK2	Telinga Parabola 22 in
ML155551321	‘anianiau	Present day	Kuhn, David	2/27/2017	Halepa'akai Stream	Sony PCM M10	Telinga PRO-8 MK2	Telinga Parabola 22 in
ML155552771	‘anianiau	Present day	Paxton, Kristina	2/27/2017	Halepa'akai Stream	Marantz PMD 661	Sennheiser MKH 20	
ML156006261	‘akeke‘e	Present day	Kuhn, David	6/9/2010	Pihea Trail in Koke‘e State Park	Sony PCM M10	Telinga PRO-8 MK2	Telinga Parabola 22 in
ML155989971	‘akeke‘e	Present day	Kuhn, David	2/29/2012	Pihea Trail in Koke‘e State Park	Sony PCM M10	Telinga PRO-8 MK2	Telinga Parabola 22 in
ML155999911	‘akeke‘e	Present day	Kuhn, David	3/14/2012	Pihea Trail in Koke‘e State Park	Sony PCM M10	Telinga PRO-8 MK2	Telinga Parabola 22 in
ML155998361	‘akeke‘e	Present day	Kuhn, David	3/14/2012	Pihea Trail in Koke‘e State Park	Sony PCM M10	Telinga PRO-8 MK2	Telinga Parabola 22 in
ML155991721	‘akeke‘e	Present day	Kuhn, David	3/28/2014	Pihea Trail in Koke‘e State Park	Sony PCM M10	Telinga PRO-8 MK2	Telinga Parabola 22 in
ML155981381	‘akeke‘e	Present day	Hite, Justin	3/28/2015	Halepa'akai Stream	Marantz PMD 661	Sennheiser MKH 20	Telinga Parabola 15.5 in
ML155975631	‘akeke‘e	Present day	Hite, Justin	4/2/2015	Halepa'akai Stream	Marantz PMD 661	Sennheiser MKH 20	Telinga Parabola 15.5 in
ML155967451	‘akeke‘e	Present day	Hite, Justin	5/13/2015	Halepa'akai Stream	Marantz PMD 661	Sennheiser MKH 20	Telinga Parabola 15.5 in
ML155612031	‘akeke‘e	Present day	Kuhn, David	5/13/2016	Pihea Trail in Koke‘e State Park	Sony PCM M10	Telinga PRO-8 MK2	Telinga Parabola 22 in
ML155553061	‘akeke‘e	Present day	Kuhn, David	2/25/2017	Halepa'akai Stream	Sony PCM M10	Telinga PRO-8 MK2	Telinga Parabola 22 in

ML155553091	‘akeke‘e	Present day	Kuhn, David	2/25/2017	Halepa'akai Stream	Sony PCM M10	Telinga PRO-8 MK2	Telinga Parabola 22 in
ML155553101	‘akeke‘e	Present day	Kuhn, David	2/25/2017	Halepa'akai Stream	Sony PCM M10	Telinga PRO-8 MK2	Telinga Parabola 22 in
ML155551701	‘akeke‘e	Present day	Kuhn, David	2/26/2017	Halepa'akai Stream	Sony PCM M10	Telinga PRO-8 MK2	Telinga Parabola 22 in
