# Supplementary material for: Loss of cultural song diversity and the convergence of songs in a declining Hawaiian forest bird community
Source: R Soc Open Sci. 2019 Aug 14;6(8):190719. doi: 10.1098/rsos.190719 (PMC6731710; doi:10.1098/rsos.190719)
Supplement: Table S2 [file rsos190719supp3.pdf]

Supplemental Document Table S2

Table S2. Sample size of high-quality recordings of individually identified Kaua‘i ‘amakihi, ‘anianiau, and ‘akeke‘e during each time period.

Species	1970s	Early 2000s	Present day
Kaua‘i ‘amakihi	16	6	12
‘anianiau	8	9	9
‘akeke‘e	1	12	13
