# Supplementary material for: Loss of cultural song diversity and the convergence of songs in a declining Hawaiian forest bird community
Source: R Soc Open Sci. 2019 Aug 14;6(8):190719. doi: 10.1098/rsos.190719 (PMC6731710; doi:10.1098/rsos.190719)
Supplement: Table S3 [file rsos190719supp4.pdf]

Supplemental Document Table S3

Table S3. Principal component loading values of 11 acoustic variables for a reduced Kaua‘i ‘amakihi dataset that includes only 6 randomly chosen recordings from each time period. Acoustic characteristics with the strongest PCA loadings (>0.35) are in bold.

PC Axis	Low Freq	High Freq	Peak Freq	Song length	Trill rate	Freq Bandwidth	Total no. syllables	No. unique syllables	ln(No. notes per syllable)	ln(No. freq changes (syllable))	No. freq changes (song)	Eigenvalue	Proportion of Variation	Cumulative Proportion
PC1	**0.37**	**-0.43**	--	--	0.30	**-0.48**	0.28	-0.25	-0.24	**-0.38**	-0.14	3.84	0.35	0.35
PC2	-0.26	-0.11	**0.50**	**0.41**	-0.27	--	0.16	**-0.39**	0.18	--	**-0.47**	2.79	0.25	0.60
PC3	0.19	--	0.26	**-0.59**	0.23	-0.14	**-0.39**	-0.20	0.33	0.31	-0.28	1.31	0.20	0.80
PC4	-0.26	**-0.38**	--	-0.12	**-0.51**	-0.19	**-0.58**	--	-0.32	-0.21	--	1.26	0.11	0.91
