# Supplementary material for: Loss of cultural song diversity and the convergence of songs in a declining Hawaiian forest bird community
Source: R Soc Open Sci. 2019 Aug 14;6(8):190719. doi: 10.1098/rsos.190719 (PMC6731710; doi:10.1098/rsos.190719)
Supplement: Table S4 [file rsos190719supp5.pdf]

Supplemental Document

Table S4. Post-hoc pair-wise comparisons for a reduced Kauaʻi ʻamakihi dataset that includes only 6 randomly chosen recordings from each time period. Post-hoc comparisons test for 1) differences in acoustic characteristics among time periods based on a PERMANOVA, and 2) variability in acoustic characteristics within time periods based on a PERMDISP.

Comparison	Differences among time periods	Variability within time periods
1970s vs Early 2000s	0.84	0.70
1970s vs Present day	**0.009**	**0.04**
Early 2000s vs Present day	0.52	**0.008**
