# Supplementary material for: Loss of cultural song diversity and the convergence of songs in a declining Hawaiian forest bird community
Source: R Soc Open Sci. 2019 Aug 14;6(8):190719. doi: 10.1098/rsos.190719 (PMC6731710; doi:10.1098/rsos.190719)
Supplement: Table S5 [file rsos190719supp6.pdf]

Supplemental Document Table S5

Table S5. Principal component loading values of 11 acoustic variables for Kaua‘i ‘amakihi, ‘anianiau, and ‘akeke‘e recorded during 3 time periods over a 40-year time frame. Acoustic characteristics with the strongest PCA loadings (>0.35) are in bold.

Species	Low Freq	High Freq	Peak Freq	Song length	Trill rate	Freq Bandwidth	Total no. syllables	No. unique syllables	ln(No. notes per syllable)	ln(No. freq changes (syllable))	No. freq changes (song)	Eigenvalue	Proportion of Variation	Cumulative Proportion
Kaua‘i ‘amakihi														
PC1	0.31	**-0.47**	--	--	0.24	**-0.53**	0.27	-0.25	-0.21	**-0.37**	-0.16	2.84	0.26	0.26
PC2	0.28	0.23	**-0.42**	-0.28	**0.48**	--	0.21	0.30	--	--	**0.42**	2.47	0.22	0.48
PC3	-0.29	0.14	--	**0.62**		0.24	**0.48**	--	-0.19	**-0.36**	0.23	1.76	0.16	0.64
PC4	-0.14	-0.28	-0.16	--	**-0.36**	-0.17	**-0.40**	**0.44**	**-0.46**	-0.23	0.31	1.58	0.14	0.78
‘anianiau														
PC1	0.22	**-0.38**	-0.13	0.29	**0.43**	**-0.42**	**0.44**	--	-0.18	**-0.35**	--	3.64	0.33	0.33
PC2	0.12	-0.17	0.15	--	--	-0.19	--	**-0.50**	**0.45**	0.33	**-0.58**	2.19	0.20	0.53
PC3	0.26	-0.12	**0.46**	**-0.56**	0.19	-0.20	**-0.36**	-0.24	-0.34	-0.12	--	1.60	0.15	0.68
PC4	0.24	**0.54**	--	--	--	**0.40**	0.19	**-0.45**	-0.24	-0.34	-0.26	1.05	0.10	0.78
‘akeke‘e														
PC1	--	**0.36**	--	--	**-0.37**	**0.36**	-0.31	**0.37**	**-0.45**	**-0.39**	0.14	3.26	0.30	0.30
PC2	0.25	-0.23	--	**-0.38**	-0.30	-0.34	**-0.50**	-0.32	--	-0.14	**-0.37**	2.03	0.18	0.48
PC3	**-0.55**	-0.19	**-0.53**	0.33	**-0.35**	--	--	-0.24	-0.2	0.19	--	1.68	0.15	0.63
PC4	--	**0.48**	-0.11	-0.29	--	**0.48**	-0.19	-0.20	0.31	**0.48**	-0.23	1.38	0.13	0.76
PC5	**0.35**	0.25	--	**0.54**	-0.14	--	0.29	-0.18	--	-0.21	**-0.57**	1.10	0.10	0.86
